# Insights into circular RNAs: Biogenesis, function and their regulatory roles in cardiovascular disease

**DOI:** 10.1111/jcmm.17734

**Published:** 2023-04-01

**Authors:** Chen Ding, Yafeng Zhou

**Affiliations:** ^1^ Department of Cardiology, Dushu Lake Hospital Affiliated to Soochow University, Medical Center of Soochow University Suzhou Dushu Lake Hospital Suzhou Jiangsu China; ^2^ Institute for Hypertension of Soochow University Suzhou Jiangsu China; ^3^ Jiangsu Engineering Laboratory of Novel Functional Polymeric Materials Soochow University Suzhou Jiangsu 215123 China

**Keywords:** biogenesis, cardiovascular disease, circular RNA, function, mechanism

## Abstract

As a distinctive member of the noncoding RNA family, circular RNAs (circRNAs) are generated from single‐stranded, covalently closed structures and are ubiquitous in mammalian cells and tissues. Due to its atypical circular architecture, it was conventionally deemed insignificant dark matter for a prolonged duration. Nevertheless, studies conducted over the last decade have demonstrated that this abundant, structurally stable and tissue‐specific RNA has been increasingly relevant in diverse diseases, including cancer, neurological disorders, diabetes mellitus and cardiovascular diseases (CVDs). Therefore, regulatory pathways controlled by circRNAs are widely involved in the occurrence and pathological processes of CVDs through their function as miRNA sponges, protein sponges and protein scaffolds. To better understand the role of circRNAs and their complex regulatory networks in CVDs, we summarize current knowledge of their biogenesis and function and the latest research on circRNAs in CVDs, with the hope of paving the way for the identification of promising biomarkers and therapeutic strategies for CVDs.

## 1 INTRODUCTION

Cardiovascular diseases (CVDs) remain the leading cause of death and rising health expenditure globally.^1^ The overall prevalence of cardiovascular disease has nearly doubled from 271 million in 1990 to 550 million in 2019, and cardiovascular deaths have also surged by 6.5 million in those decades.^2^, ^3^ Meanwhile, the social burden caused by the morbidity and disability of CVDs cannot be overlooked.^4^ The globalization trend of aging and population growth has resulted in increased morbidity and mortality of CVDs in the elderly.^5^ Therefore, the identification of novel predictive biomarkers and therapeutic targets for CVDs is of paramount importance.

Of the entire human genome, only about 2% can code for proteins. Most of the remaining noncoding genomes that do not code for any proteins have now been demonstrated to be critical regulators of CVDs.^6^, ^7^, ^8^, ^9^ As a particular class of noncoding RNA, circular RNA (circRNA), initially discovered in plant viroids in 1976, has a single‐stranded, covalently closed RNA structure.^10^ Unlike linear RNAs, circRNAs are generated by covalent reverse splicing of pre‐mRNAs and are considered by‐products of linear RNAs. In the past few years, circRNAs were rarely detected owing to the lack of canonical 5′ caps and 3′ poly‐A structures.^11^ With the advent of high‐throughput sequencing and circRNA‐specific computational tools, the enigma of circRNA was gradually unravelled and became a hotpot in the noncoding RNA family.^12^, ^13^, ^14^, ^15^ Significantly, emerging studies have supported the vital role of circRNAs in CVDs, such as atherosclerosis (AS), myocardial infarction (MI), heart failure (HF), diabetic cardiomyopathy (DCM), hypertension, cardiac hypertrophy and fibrosis. A comprehensive summary of these dysregulated circRNAs would assist in elucidating the association between circRNAs and the occurrence and progression of CVDs.

Here, we review the biogenesis, structural characteristics and biological functions of circRNAs, summarize the latest research progress of circRNAs in the pathogenesis of CVDs and discuss the application prospects of circRNAs in the prevention, diagnosis and treatment of CVDs.

## 2 BIOGENESIS

**FIGURE 1:**
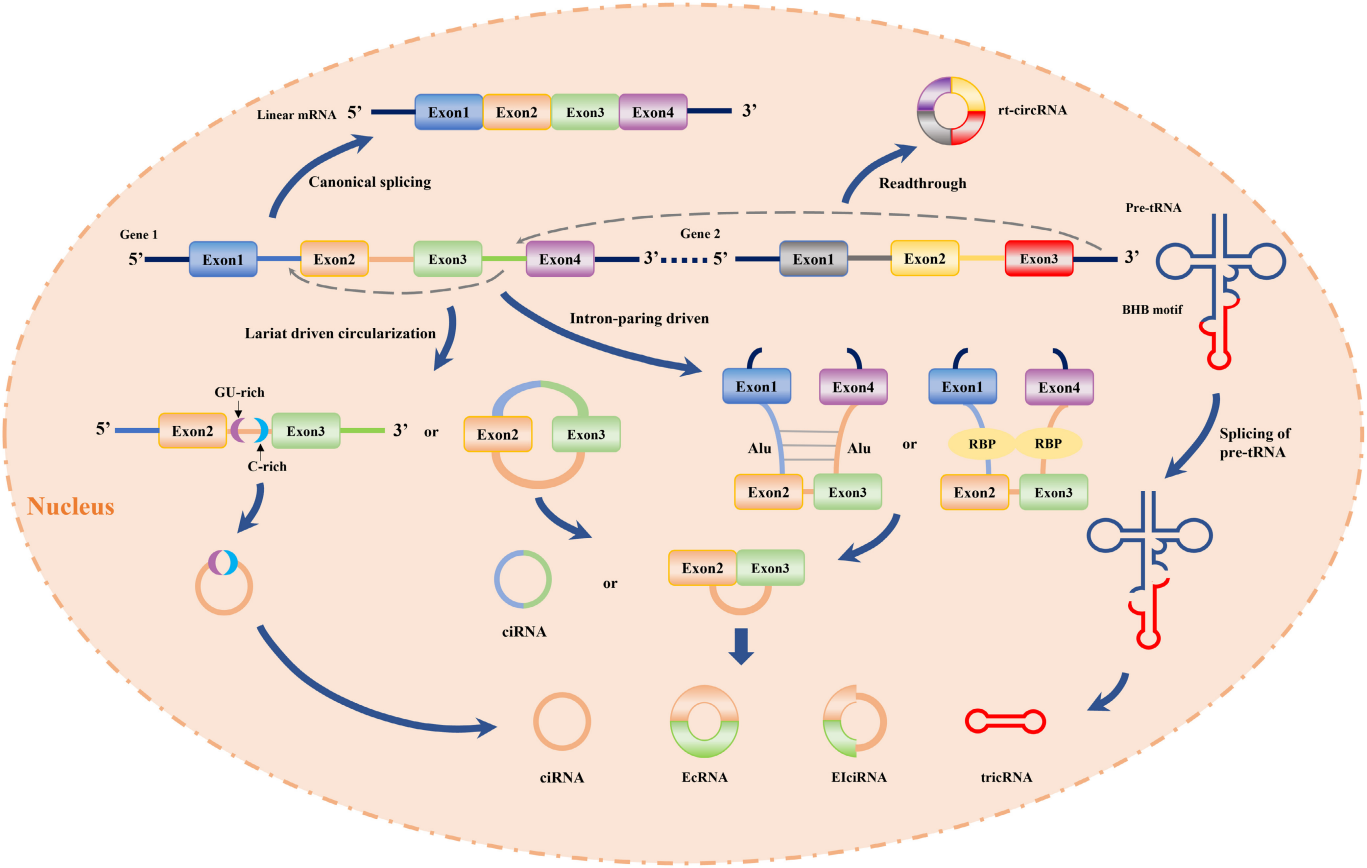
The biogenesis models of circRNAs.

In eukaryotes, spliceosomes typically snip off introns in messenger RNA (mRNA) to produce linear RNA. In contrast to this canonical splicing, circRNA undergoes a distinct form of splicing called back‐splicing, whereby the downstream 3′ splice site is connected to the upstream 5′ splice site through a 3′‐5′ phosphodiester bond and forms a covalently linked closed loop (Figure 1).^16^, ^17^, ^18^ This process is regulated by RNA‐binding proteins (RBPs), transcription factors, cis‐acting elements and trans‐acting factors, and there exists a competitive relationship between canonical splicing and back‐splicing.^19^, ^20^

Jeck proposed two back‐splice formation models of circRNA in 2013^12^: the lariat‐driven circularization model (also called the exon‐skipping model) and the intron‐paring‐driven model. In the first model, exon skipping generates a functional transcript and forms a lariat intermediate composed of the skipped exons and introns. This lasso can undergo secondary cleavage to release exon–intron circular RNAs (EIciRNAs) containing both introns and exons, exonic circRNAs (EcRNAs) containing only exons or intron‐only circRNAs (ciRNAs).^21^, ^22^ When the 7‐nt GU‐rich base sequence and the 11‐nt C‐rich base occur near the 5′ splice site and branch site sequence, those motifs can induce introns to form lariat structures to avoid degradation by branching enzymes, resulting in stable ciRNAs.^23^

If we regard the first model as a passive manner of circNRA formation, the second model is more like an active process of direct splicing to form circRNAs after intron pairing. In the intron pairing‐driven circularization model, direct base pairing (such as Alu repetitive elements) in long flanking introns can generate the formation of secondary structures in the pre‐mRNA, which is crucial for the subsequent connection between downstream splice donors and upstream splice acceptors.^11^, ^24^ Several studies have demonstrated that some RBPs can recognize and bind specific sequences within flanking introns, bring two splicing sites close enough to form a loop and then enhance circRNA biogenesis.^25^, ^26^ For example, the alternative splicing factor Quaking (QKI) and Muscle blind protein (MBL) can promote the efficiency of circRNA production through protein‐to‐protein interactions or self‐dimerization.^16^, ^26^ Additionally, downregulation of MBL leads to a significant decrease in circMbl production, which further illustrates the crucial regulatory role of RBPs in circRNA cyclization.

Furthermore, a small fraction of circRNAs originate from pre‐transfer RNA (pre‐tRNA). The intronic elements of tRNAs must be removed to form functional RNAs. In this process, the tRNA splicing endonuclease (TSEN) complex recognizes the bulge‐helix‐bulge (BHB) motif and cleaving within the anticodon loop of pre‐tRNA. Subsequently, the cleaved introns are joined together by RtcB‐like proteins to form tRNA intronic circular RNAs (tricRNAs).^27^, ^28^ Moreover, a recent study identified a novel type of circular transcript: read‐through circRNA (rt‐circRNA), which consists of two adjacent genes on the same strand and accounts for a small portion of all circRNAs.^29^

Within the nucleus, pre‐mRNA removes introns by canonical splicing to form linear mRNA. Linear splicing produces circRNAs through two back‐splice formation models: the lariat‐driven circularization model and the intron‐paring‐driven model. These two ways can eventually form ciRNA, EcRNA or EIciRNA. Furthermore, two special circRNAs, tricRNAs and rt‐circRNA, can be formed through pre‐tRNA cleavage and two adjacent genes. mRNA: messenger RNA; EIciRNAs: exon–intron circRNA; EcRNAs: exonic circRNAs; ciRNAs: intronic circRNA; tricRNAs: tRNA intronic circular RNAs; rt‐circRNA: read‐through circRNA; pre‐tRNA: pre‐transfer RNA; RBPs: RNA‐binding proteins; BHB: bulge‐helix‐bulge.

## 3 CHARACTERISTICS

CircRNAs are abundantly present in a variety of organisms such as Drosophila, nematodes, plants and mammals. In mammals, including humans, mice and rats, circRNAs are highly conserved.^30^, ^31^ It has been reported that approximately 9% of cardiac‐expressed genes can produce circRNAs.^13^, ^30^, ^32^ A recent RNA sequencing analysis has revealed that about 30% circRNAs in the heart are conserved between mice and rats, and approximately 10% are conserved among the three species.^33^ Moreover, circRNAs can avoid degradation by PNase R enzyme and other exonucleases due to their unique circular structure and maintain a stable structure.^34^ Hence, circRNA has a longer half‐life than linear RNA, with an average half‐life of about five times that of mRNA.^12^, ^35^ Additionally, the expression of circRNAs is also tissue‐specific, and it can be expressed in normal tissues of the colon, heart, kidney, liver, lung, stomach and human gland.^31^, ^36^, ^37^ Meanwhile, the abundance of circRNAs is negatively correlated with the proliferative capacity of cells.^36^ For instance, low proliferative cells such as cardiomyocytes usually have higher expression levels of circRNAs. These unique features make circRNAs have great potential to be ideal biomarkers for CVDs.

## 4 FUNCTION

**FIGURE 2:**
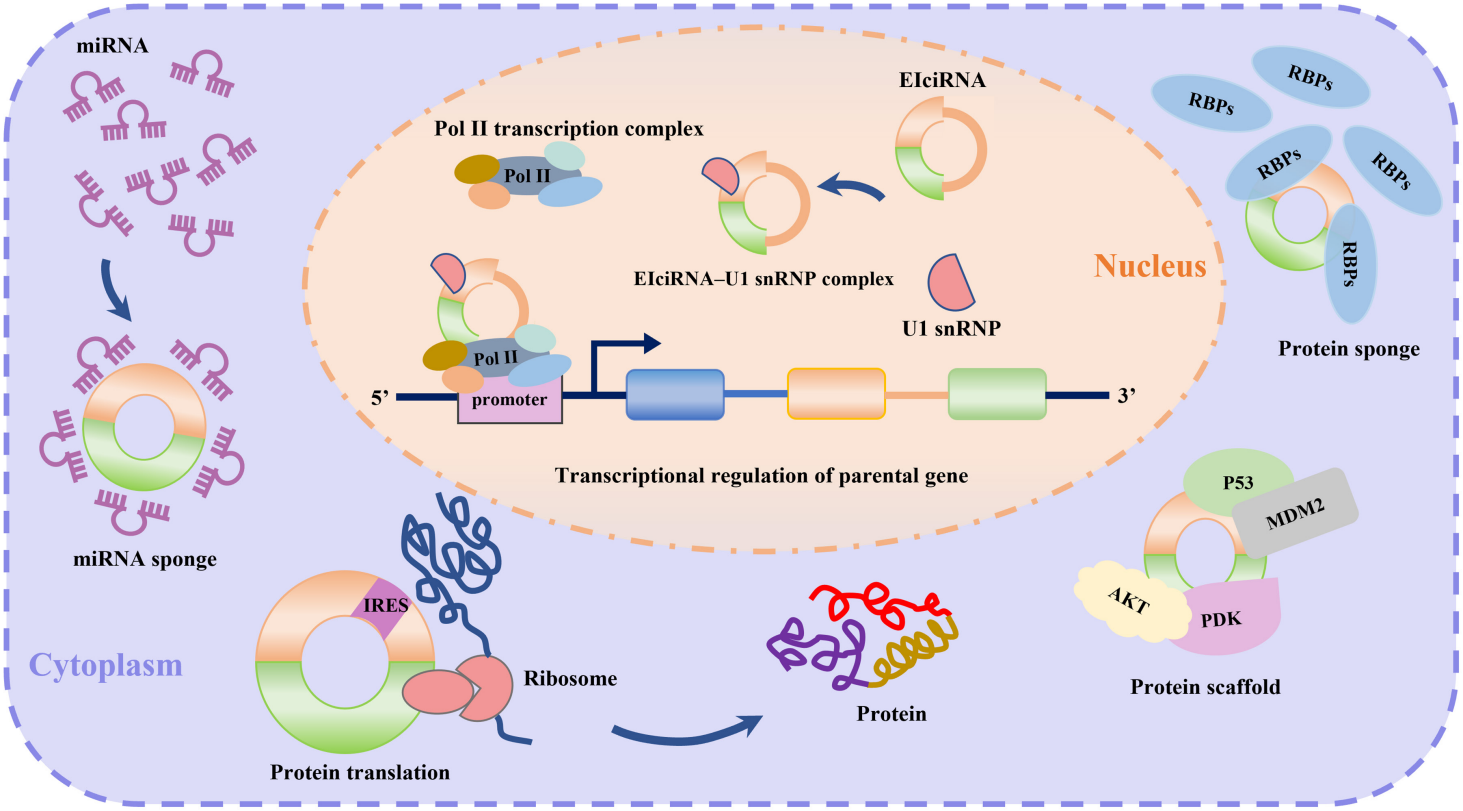
Biological functions of circRNAs.

Initially, circRNAs were considered noncoding RNA molecules with regulatory functions. Nevertheless, recent studies have unveiled several additional roles of circRNAs, as depicted in Figure 2. By regulating mRNA and protein expression, circRNAs indirectly regulate target genes. Moreover, circRNAs can act as protein scaffolds, thereby facilitating protein–protein interactions. Furthermore, a subset of circRNAs has been shown to mediate cap‐independent translation, while circRNAs located in the nucleus can also regulate the transcription of parental genes. In the following module, we will systematically review the biological functions of circRNAs.

## 5 miRNA SPONGE

MicroRNAs (miRNAs) are a class of endogenous ~23 nucleotide RNAs that regulate post‐transcriptional silencing of target genes by pairing to the mRNAs of protein‐coding genes.^38^ As competing endogenous RNAs (ceRNAs), circRNAs can act as decoys for miRNA by binding to miRNA response elements (MREs) and subsequently hindering miRNA activity, thus regulating mRNA expression.^39^, ^40^ This miRNA sponge function of circRNAs has been demonstrated in several studies. For example, the cerebellar degeneration‐related protein 1 antisense (Cdr1as) contains 63 conserved binding sites for the miRNA miR‐7.^13^, ^41^ In neural tissues, CDR1as can bind to miR‐7 and increase miR‐7 target mRNA levels by inhibiting miR‐7 activity.^13^ Similarly, by acting as a sponge for miR‐7, CDR1as promotes MI by targeting miR‐7 and its downstream factors, SP1 and polymerase (PARP).^42^ Additionally, circCHFR regulates cell growth, migration and inflammation in oxidized low‐density lipoprotein (ox‐LDL)‐treated human vascular smooth muscle cells (VSMCs) by functioning as a sponge of miR‐214‐3p.^43^ Furthermore, circUSP36 aggravates endothelial cell dysfunction in AS caused by ox‐LDL by competitively binding to miR‐637.^44^ These researchers indicate the crucial role of circRNAs in the pathological development of CVDs via the miRNA sponge effect.

## 6 PROTEIN SPONGE

In addition to their function as miRNA sponges, researchers have demonstrated that circRNAs can bind to specific protein binding sites, thereby playing the role of protein sponges. RBPs are a class of proteins involved in gene transcription and translation.^45^ Combining circRNAs to form circRNA‐RBP complexes can regulate the life cycle of circRNAs, protein biogenesis and transcription of parental genes.^16^, ^46^, ^47^, ^48^ For instance, circMbl and its intronic sequences flanking the second exon contain conserved MBL binding sites, and MBL can induce the circularization of circMbl through these binding sites.^16^ Similarly, circPABPN1 suppressed the binding of HuR to PABPN1 mRNA and the translation level of PABPN1 by binding to HuR in human cervical carcinoma HeLa cells.^46^ In addition, circular antisense noncoding RNA in the INK4 locus (circANRIL) regulates ribosome biogenesis and promotes atheroprotection by binding to the lysine‐rich domain of pescadillo zebrafish homologue 1 (PES1).^47^, ^48^ Compared with linear RNA, the tertiary structure of circRNAs has more extraordinary protein binding ability, which may be an essential structural foundation for the interaction between RBPs and circRNAs.^49^

## 7 PROTEIN SCAFFOLD

Some circRNAs can act as protein scaffolds to regulate gene expression and protein function by promoting the interaction of two or more proteins.^50^ For example, circ‐forkhead box protein O3 (circ‐FOXO3) can act as a protein scaffold of MDM2 and p53, facilitating MDM2‐induced p53 ubiquitination and subsequent degradation, thereby promoting tumour apoptosis.^51^ Moreover, circ‐Foxo3 can interact with cyclin‐dependent kinase 2 (CKD2) and the CDK2 inhibitor p21 to form a circ‐FoxO3‐p21‐CDK2 ternary complex and block cell cycle progression.^52^ Circ‐Amotl1 can serve as a scaffold for AKT and phosphoinositide‐dependent kinases (PDK), activate AKT phosphorylation and pAKT nuclear translocation, enhance cell proliferation and survival and protect against doxorubicin‐induced cardiomyopathy.^53^

## 8 PROTEIN TRANSLATION

Generally, translation initiation in eukaryotes relies on the cap structure at the 5′ end of the mRNA. Due to the lack of the 5′ cap, circRNAs have long been considered to have no translational function. In 1998, Perriman et al.^54^ reported that a circular mRNA containing a green fluorescent protein (GFP) open reading frame (ORF) encoded GFP in *Escherichia coli*. Subsequently, multiple studies have fully demonstrated that circRNAs can complete protein translation independent of the 5′‐cap structure, enriching people's understanding of the functions of circRNAs.^55^, ^56^, ^57^

Recent studies have shown that the translation of circRNAs is mainly mediated by two cap‐independent translation mechanisms, IRES and m6A. IRESs are sequences located in the 5′ UTRs of mRNAs that can directly recruit the 40S subunit of the ribosome and initiation factors on RNA to initiate translation.^58^ The m6A motif promotes protein translation under the combined action of YTHDF3 and initiation factor eIF4G2, and a single m6A site is sufficient to drive translation initiation.^59^ Notably, the two mechanisms of circRNA‐protein translation function are not independent of each other. High m6A methylation levels were detected in circZNF609, which translates protein via the IRES pathway.^60^ Nevertheless, further research is necessary to determine the specific relationship between these two mechanisms.

## 9 TRANSCRIPTIONAL REGULATION OF PARENTAL GENES

CiRNAs and EIciRNAs are primarily located in the nucleus, can function as transcriptional regulators to enhance the expression of parental genes in a cis‐acting manner. For example, ci‐ankrd52 can accumulate at its sites of transcription and then interact with RNA Pol II, acting as a cis‐regulator in its parental gene ANKRD52.^23^ Besides, EIciRNA circEIF3J and circPAIP2 can form an EIciRNA–U1 snRNP complex through specific RNA–RNA interactions between U1 snRNA and EIciRNA, which subsequently interact with the promoter of the RNA pol II transcription complex and enhance the expression of their parental genes EIF3J and PAIP2. Meanwhile, knockdown of circEIF3J and circPAIP2 resulted in decreased transcript levels of EIF3J and PAIP2, respectively.^61^

In the nucleus, circRNAs and U1 snRNP can form an EIciRNA–U1 snRNP complex and bind to the promoter to enhance the expression of parental genes. In the cytoplasm, circRNA can function as a miRNA sponge, protein sponge and protein scaffold. A small fraction of RNA has been demonstrated to accomplish protein transcription cap independently.

## 10 THE ROLE IN CARDIOVASCULAR DISEASES

It has been less than a decade since the first study focusing on the biological role of circRNAs in CVDs.^62^ Therefore, the relationship between circRNAs and CVDs is still a burgeoning area of research. While still in its infancy, the role of circRNAs in the occurrence and progression of CVDs has attracted much attention from researchers, and the number of related studies is rapidly increasing. In this section, we present a detailed summary of CVD‐related circRNAs and their functions to support the evaluation of circRNAs as potential biomarkers and therapeutic targets for CVDs.

## 11 ATHEROSCLEROSIS

Atherosclerosis (AS) is a chronic inflammatory disorder of the arterial vessel walls and the primary pathological basis of CVDs. Endothelial cells (ECs) and vascular smooth muscle cells (VSMCs) are crucial components of the coronary intima and media, respectively. Abnormal proliferation, migration and invasion of these two cells play a vital role in the pathogenetic process of AS.^63^, ^64^ Growing evidence suggests that circRNAs regulate cell proliferation and invasion via targeted miRNAs, thereby participating in AS progression (Table 1). For instance, the expression of circ‐0003757 showed a dose‐dependent increase in oxidative‐modified LDL (ox‐LDL)‐induced human umbilical vein endothelial cells (HUVECs). Subsequently, hsa_circ_0003575 silencing significantly promoted the proliferation and the ability to form capillary‐like structures while reducing the apoptosis of ox‐LDL‐induced HUVECs.^65^ Yang et al. validated the promoting role of circCHFR in AS via the miR‐370/Forkhead box protein O1 (FOXO1)/Cyclin D1 pathway. CircCHFR can act as a sponge for miR‐370, which targets the 3′ UTR of its downstream transcription factor FOXO1 through complementary binding sites.^66^ FOXO1, in turn, binds to the promoter region of CCND1 mRNA, promoting Cyclin D1 expression and ultimately facilitates HUVECs proliferation and migration. Stromal interaction molecule 1 (STIM1) is a vital regulator of VSMCs function.^72^ Circ‐SATB2 and circ_0029589 can target STIM1 via miR‐939 and miR‐214‐3p, respectively, to regulate the proliferation, migration and invasion of VSMCs.^67^, ^68^ Furthermore, circANRIL can induce apoptosis, inhibit proliferation and confer atheroprotection by generating nucleolar stress and p53 activation through binding to pescadillo homologue 1 (PES1).^47^

**TABLE 1:** circRNAs associated with atherosclerosis.

circRNA	Exp	Targets	Mechanism	Function	Ref
CircANRIL	↑	PES1	Induces nucleolar stress and p53 activation	Confers atheroprotection by induction of apoptosis and inhibition of proliferation	[47]
Hsa_circ_0003575	↑	Unknown	Unknown	Silencing promotes the proliferation and angiogenesis ability of HUVECs	[65]
circCHFR	↑	miR‐370	miRNA sponge	Facilitates the proliferation and migration of VSMCs via miR‐370/FOXO1/Cyclin D1 pathway	[66]
Circ‐SATB2	↑	miR‐939	miRNA sponge	Promotes proliferation and differentiation of VSMCs through miR‐939/STIM1 axis	[67]
Circ_0029589	↑	miR‐214‐3p	miRNA sponge	Knockdown inhibits the proliferation, migration and invasion of VSMCs via regulating miR‐214‐3p and STIM1	[68]
circ_0090231	↑	miR‐635	miRNA sponge	Knockdown reduces cell injury and pyroptosis through the miR‐635/NLRP3 axis	[69]
Hsa_circ_0030042	↓	eIF4A3	Protein Sponge	Inhibits abnormal autophagy and ameliorated plaque stability by obstructing the recruitment of eIF4A3 to beclin1 and FOXO1 mRNA	[70]
circDENND1B	↓	miR‐17‐5p	miRNA sponge	Inhibits atherosclerosis by promoting cholesterol efflux via miR‐17‐5p/Abca1axis	[71]

Abbreviations: Abca1, ATP binding cassette subfamily A member 1; circANRIL, circular antisense noncoding RNA in the INK4 locus; eIF4A3, eukaryotic initiation factor 4A‐III; Exp, expression; FOXO1, forkhead box protein O1; HUVECs, human umbilical vein endothelial cells; PES1, pescadillo zebrafish homologue 1; Ref, reference(s); STIM1, stromal interaction molecule 1; VSMCs, vascular smooth muscle cells.

It is worth noting that circRNAs can also affect AS progression in other ways. Treating human aortic endothelial cells (HAECs) with ox‐LDL increases the expression of circ_0090231, which was also identified as a sponge for miR‐635.^69^ Furthermore, knockdown of circ_0090231 inhibits cell apoptosis through the circ_0090231/miR‐635/NLRP3 axis, thereby inhibiting AS development. Hsa_circ_0030042 inhibits abnormal autophagy by blocking its recruitment to beclin1 and FOXO1 mRNA and protects advanced atherosclerotic plaque stability by targeting eukaryotic initiation factor 4A‐III (eIF4A3).^70^ CircDENND1B can inhibit foam cell formation by promoting the expression of ATP binding cassette subfamily A member 1 (Abca1) and participating in the anti‐atherosclerotic effect of IL‐1β monoclonal antibody (IL‐1β mAb) by promoting cholesterol efflux through the miR‐17‐5p/Abca1 pathway.^71^ Future studies will likely uncover additional ways in which circRNAs contribute to AS progression.

## 12 MYOCARDIAL INFARCTION/ISCHEMIA REPERFUSION INJURY

Myocardial infarction (MI) is a clinical syndrome resulting from a disturbance in the equilibrium between myocardial oxygen supply and demand.^73^ Typically, the coronary arteries perform an essential function by providing blood and nutritional sustenance to the heart. However, when an atherosclerotic plaque in a coronary artery ruptures and haemorrhages, it can result in stenosis and even occlusion of the artery. Prolonged ischemia and hypoxia can trigger a cascade of events such as necrocytosis, apoptosis, and the inflammatory response of cardiomyocytes, ultimately culminating in heart failure and cardiac remodelling.^74^, ^75^, ^76^, ^77^ Recent research has substantiated that several circRNAs engage in multiple pathological phases of MI through diverse pathways (Table 2).

**TABLE 2:** circRNAs associated with myocardial infarction or ischemia/reperfusion injury.

circRNA	Exp	Targets	Mechanism	Function	Ref
MICRA	↓	Unknown	Unknown	The expression level of MICRA is valuable to risk stratify MI patients	[78, 79]
Cdr1as	↑	miR‐7a	miRNA sponge	Promotes MI‐related injuries by reducing the activity of miR‐7a in inhibiting the expression of its target genes, PARP and SP1.	[42]
Cdr1as	↑	Unknown	Unknown	The expression of circRNA CDR1as in the AMI region of the porcine heart was negatively correlated with infarct size	[80]
Circ‐Ttc3	↑	miR‐15b	miRNA sponge	Ameliorates MI‐induced cardiomyocyte apoptosis through circ‐Ttc3/miR‐15b/Arl2 pathway	[81]
CircRNA 010567	↑	miR‐141	miRNA sponge	CircRNA 010567‐siRNA mitigates hypoxia‐induced cardiomyocyte injury via miR‐141/DAPK1 axis	[82]
circSNRK	↓	miR‐103‐3p	miRNA sponge	Overexpression of circSNRK targets miR‐103‐3p to reduce apoptosis and promotes cardiac regeneration through GSK3β/β‐catenin pathway	[83]
Circ ACAP2	↑	miR‐29	miRNA sponge	Promotes apoptosis after MIn by sponging miR‐29	[84]
Circ_0068655	↑	miR‐498	miRNA sponge	Promotes apoptosis and suppressed cell viability by sponging miR‐498 and upregulating PAWR expression	[85]
CircSAMD4A	↑	miR‐138‐5p	miRNA sponge	Promotes cardiomyocytes apoptosis and inflammatory response by targeting miR‐138‐5p	[86]
ACAP2	↑	Unknown	Unknown	Induces the maturation of miR‐532 and the apoptosis of cardiomyocyte	[87]
MFACR	↑	miR‐125	Increases methylation of miR‐125 gene	Promotes cardiomyocyte apoptosis induced by hypoxia	[88]
CircNfix	↑	miR‐214	miRNA sponge	Inhibits cardiac regenerative repair after MI by promoting Ybx1 degradation and decreasing miR‐214 activity	[89]
CircFndc3b	↓	FUS	Protein Sponge	Overexpression promotes neovascularization and cardiac repair after MI via FUS/VEGF‐A signal pathway	[90]
CircCDYL	↓	miR‐4793‐5p	miRNA sponge	Promotes myocardial regeneration and repair after MI through sponging miR‐4793‐5p	[91]
circHipk3	Unknown	miR‐133a	miRNA sponge	Decreases the size of scar area and promotes regenerative repair after MI	[92]
circSamd4	Unknown	VCP	promotes the mitochondrial translocation of Vcp	Reduces infarct area and restores cardiac function after MI through circSamd4/Vcp/Vdac1 pathway	[93]
ACR	↓	DNMT3B	Protein Sponge	Inhibits DNA methylation of Pink1 by binding to DNMT3B and subsequently improves I/R injury by ameliorating autophagy through the ACR/Pink1/FAM65B regulatory axis	[94]
circFoxo3	↓	KAT7	circRNA‐protein interaction	Attenuate myocardia I/R injury by suppressing autophagy by targeting KAT7/HMGB1 pathway	[95]
circRbms1	↑	miR‐92a	miRNA sponge	Inhibits the progress of I/R injury by regulating circRbms1/miR‐92a/BCL2L11 signalling pathway	[96]
Circ‐NNT	↑	miR‐33a‐5p	miRNA sponge	Promotes myocardial I/R injury through activating pyroptosis via circ‐NNT/miR‐33a‐5p/USP46 axis	[97]

Abbreviations: ACR, autophagy‐related circular RNA; AMI, acute myocardial infarction; Cdr1as, cerebellar degeneration‐related protein 1 antisense; DAPK1, death‐associated protein kinase 1; FAM65B, family with sequence similarity 65 member B; FUS, fused in sarcoma; HMGB1, high mobility group box 1; I/R injury, ischemia/reperfusion injury; MI, myocardial infarction; MICRA, myocardial infarction‐associated circular RNA; PARP, polymerase; PAWR, PRKC apoptosis WT1 regulator; Pink1, phosphatase and tensin homologue‐induced putative kinase 1; Tct3, tetratricopeptide repeat domain 3; USP46, ubiquitin‐specific protease‐46; VEGF‐A, vascular endothelial growth factor‐A; Ybx1, Y‐box binding protein 1.

Vausort et al. were the pioneers in demonstrating that myocardial infarction‐associated circular RNA (MICRA) expression levels were lower in blood samples from MI patients than in healthy volunteers. Besides, patients with low MICRA levels were at relatively higher risk for left ventricular (LV) dysfunction 3–4 months after MI.^78^ The association of MICRA with the degree of LV dysfunction after acute MI and the inverse relationship between MICRA levels and ejection fraction were subsequently reconfirmed in another study by the same research group. Thus, it was suggested that MICRA could be helpful in the risk stratification of MI patients.^79^ Furthermore, three subsequent studies confirmed that circular tetratricopeptide repeat domain 3 (circ‐Ttc3), circRNA 010567 and circSNRK could sponge specific miRNAs and play a vital protective role in MI by inhibiting rat cardiomyocyte apoptosis.^81^, ^82^, ^83^ In contrast, several other circRNAs, including ACAP2, circ_0068655, circSAMD4A and MFACR, promote cardiomyocyte apoptosis through their interaction with miRNAs.^84^, ^85^, ^86^, ^87^, ^88^

Furthermore, Geng et al. demonstrated that the Cdr1as/miR‐7 pathway in mouse brains also exists in cardiomyocytes. Meanwhile, Cdr1as overexpression was proven to increase cardiac infarct size in MI mice by inhibiting miR‐7 and its downstream targets PARP and SP1, as well as aggravating cardiomyocyte apoptosis under hypoxia treatment.^42^ However, in a recent study, Cdr1as was found to reduce MI size and improve cardiac function after MI.^80^ This study found that the expression of Cdr1as in the AMI region of domestic pig hearts was significantly increased, and there was a significant positive correlation between Cdr1as and LV, as well as right ventricular ejection fraction (LVEF) and LV stroke volume, and negatively correlated with infarct size. The discrepant results between the two studies may be attributed to differences in the experimental species and AMI intervention. Overall, existing studies have confirmed that circRNAs regulate MI progression, but the exact role of CDr1as remains controversial and deserves further investigation in future studies.

Opening a blocked coronary artery and promptly restoring blood perfusion to myocardial cells, known as reperfusion, is the primary method of myocardial cell resuscitation in clinical practice. However, reperfusion is a double‐edged sword. While saving dying cardiomyocytes, calcium load and a large number of reactive oxygen species can also aggravate cardiomyocyte damage.^98^ Inhibition of autophagy during ischemia/reperfusion (I/R) is beneficial for protecting cardiomyocytes.^99^ Recent studies have proven that circRNAs can participate in the regulation of I/R injury through autophagy. Autophagy‐related circular RNA (ACR) can inhibit the methylation of the phosphatase and tensin homologue‐induced putative kinase 1 (Pink1) promoter. Then, the family with sequence similarity 65 member B (FAM65B) can be phosphorylated by Pink1 at S46, thereby alleviating I/R injury by suppressing autophagy and cell death.^94^ Similarly, circular RNA Foxo3 relieves I/R injury by repressing autophagy via the KAT7/high mobility group box 1 (HMGB1) axis.^95^ Jin et al. demonstrated increased expression of circRbms1 in I/R mice and H9C2 cells, and subsequent circRbms1 knockdown experiments confirmed that silencing circRbms1 could reduce myocardial injury and inhibit H2O2‐induced cardiomyocyte apoptosis after I/R by targeting Mir‐92a.^96^ In addition to autophagy, a recent study showed that circRNAs could regulate I/R injury through pyroptosis.^97^ In contrast to the sham group, the protein expression of pyroptosis‐related factors increased in the hearts of I/R mice in a dose‐dependent manner. The results of luciferase reporter assays and gene overexpression/knockdown studies suggest that circ‐NNT activates pyroptosis and promotes I/R injury by acting as a sponge for miR‐33a‐5p in cardiomyocytes and subsequently directly modulates ubiquitin‐specific protease‐46 (USP46). In addition, whether circRNAs regulate IR through other pathways remains to be further investigated.

In addition to the aforementioned aspects, researchers have been keen on exploring the role of circRNAs in post‐MI prognosis. Recent studies have confirmed that five circRNAs regulate cardiac regenerative repair after MI.^89^, ^90^, ^91^, ^92^, ^93^ CircNfix acts as a miR‐214 sponge to negatively regulate cardiomyocyte proliferation around infarcted myocardium by promoting Y‐box binding protein 1 (Ybx1) ubiquitination and degradation.^89^ Therefore, downregulation of circNfix can promote angiogenesis while inhibiting cardiomyocyte apoptosis, thereby significantly reducing the fibrotic scar area. Overexpression of circFndc3b promotes endothelial cell angiogenesis through the fused in sarcoma (FUS)/vascular endothelial growth factor‐A (VEGF‐A) axis, inhibits endothelial cell apoptosis, shrinks infarct size and ultimately improves LV function after MI.^90^ Overexpression of circCDYL and circHipk3 can both promote the proliferation of cardiomyocytes and promote cardiac regeneration in newborn and adult mice, respectively.^91^, ^92^ Similarly, circSamd4 reduces mitochondrial oxidative stress and promotes cardiomyocyte proliferation, thereby reducing the area of fibrosis and promoting cardiac repair after MI.^93^ These studies collectively suggest that circRNAs may be potential targets for improving MI prognosis.

## 13 CARDIAC HYPERTROPHY/CARDIAC FIBROSIS/HEART FAILURE

Both cardiac hypertrophy and fibrosis are closely related to heart failure. When the heart is exposed to various physiological or pathological stimuli, it undergoes adaptive hypertrophy to maintain its normal function. However, persistent cardiac hypertrophy led to cardiac dysfunction and gradually progressed to heart failure. Cardiac fibrosis is one of the most frequent causes of cardiac dysfunction. Excessive deposition of extracellular matrix (ECM) disorganizes the structure of cardiomyocytes and reduces the compliance of cardiac tissue, which further accelerates the progression of heart failure.

Several studies have demonstrated that circRNAs are dysregulated in cardiac hypertrophy (Table 3). Yang et al.^113^ revealed that 89 circRNAs were upregulated in the left ventricle of cardiac hypertrophy mice compared to controls, while 30 were downregulated. Meanwhile, most of these altered genes were involved in metabolism, response to stimulation and biological regulation. These results suggest that altered circRNAs may play a role in stimulating cardiomyocyte hypertrophy through these processes. Additionally, circRNAs can act as miRNA sponges to regulate cardiac hypertrophy.^62^, ^100^ MiR‐223 positively regulates cardiac hypertrophy through apoptosis repressor with caspase recruitment domain (ARC). Heart‐related circRNA (HRCR) can sponge miR‐223 and counteract the inhibition of ARC expression and activity by miR‐223, ultimately suppressing cardiac hypertrophy and heart failure.^62^ Similarly, as an endogenous sponge of miR‐133a in cardiomyocytes, knockdown and overexpression of circSlc8a1 could ameliorate and aggravate cardiac hypertrophy, respectively.^100^ Two recent studies have shown that circNfix and RNA circ_0001006 can alleviate and promote myocardial hypertrophy in mice by targeting the miR‐145‐5p/activating transcription factor 3 (ATF3) and miR‐214‐3p/PAK6 pathways, respectively.^103^, ^104^ Lavenniah et al.^114^ took advantage of the properties of circRNAs and constructed artificial circRNA sponges (circmiRs) in vitro to target miR‐132 and miR‐212, which drive cardiac hypertrophy and attenuate the progression of cardiac hypertrophy caused by pressure overload. Moreover, circRNAs can interact with several miRNAs simultaneously. CircRNA_000203 was significantly upregulated in cardiomyocytes of mice with cardiac hypertrophy and overexpression of circRNA_000203 aggravated cardiac hypertrophy in mice.^101^ A dual‐luciferase reporter assay showed that circRNA_000203 contained binding sites that could interact with these two miRNAs. Mechanistically, circRNA_000203 increased the level of the prohypertrophic transcription factor Gata4 in cardiomyocytes by inhibiting Mir‐26b‐5p and Mir‐140‐3p, thereby aggravating cardiac hypertrophy. Another circRNA, circ‐sirt1, acts as a sponge for miR‐3681‐3p and miR‐5195‐3p to upregulate the expression of sirtuin 1 (SIRT1), promote autophagy and then inhibit cardiac hypertrophy.^102^ These studies have confirmed the vital role of circRNAs in cardiac hypertrophy.

**TABLE 3:** circRNAs associated with cardiac hypertrophy, cardiac fibrosis or heart failure.

circRNA	Exp	Targets	Mechanism	Function	Ref
HRCR	↓	miR‐223	miRNA sponge	Inhibits cardiac hypertrophy and HF by targeting the miR‐223/ARC pathway	[62]
circSlc8a1	↓/−	miR‐133a	miRNA sponge	Inhibits pathological hypertrophy by sequestering miR‐133a	[100]
circRNA_000203	↑	miR‐26b‐5p、miR‐140‐3p	miRNA sponge	Exacerbates cardiac hypertrophy via sponging miR‐26b‐5p and miR‐140‐3p	[101]
circ‐SIRT1	↓	miR‐3681‐3p/miR‐5195‐3p	miRNA sponge	Promotes autophagy and inhibits CH through activating SIRT1	[102]
circNfix	↓	miR‐145‐5p	miRNA sponge	Attenuates overload‐induced CH through miR‐145‐5p/ATF3 axis	[103]
circ_0001006	↑	miR‐214‐3p	miRNA sponge	Aggravates CH by MiR‐214‐3p/PAK6 pathway	[104]
CircNFIB	↓	miR‐433	miRNA sponge	Inhibits cardiac fibroblast proliferation by targeting miR‐433	[105]
circHIPK3	↑	miR‐29b‐3p	miRNA sponge	Aggravates angiotensin II‐induced cardiac fibrosis through the circHIPK3/miR‐29b‐3p pathway	[106]
circYap	↓	ACTG, TPM4	Protein scaffold	Relieves cardiac fibrosis via binding with TMP4 and ACTG	[107]
circNlgn	Unknown	H2AX	Unknown	Mediates doxorubicin‐induced cardiac remodelling and fibrosis	[108]
CDR1as	↑	miR‐135a, miR‐135b	miRNA sponge	Regulates the occurrence and progression of HF through the miR135a/HMOX1 and miR‐135b/HMOX1 axes	[109]
circ‐HIPK3	↑	miR‐17‐3p	miRNA sponge	Aggravate HF through miR‐17‐3p–ADCY6 axis	[110]
circ_0062389	↑	TGF‐β1	Unknown	Knockdown alleviates cardiomyocyte apoptosis in rats via TGF‐β1/Smad3 signalling pathway	[111]
circSnx12	↓	miR‐224‐5p	miRNA sponge	Regulates ferroptosis and oxidative stress during HF by sponging miR‐224‐5p	[112]

Abbreviations: ACTG, gamma‐actin; ADCY6, adenylate cyclase type 6; ARC, apoptosis repressor with caspase recruitment domain; ATF3, activating transcription factor 3; CH, cardiac hypertrophy; EGR, early growth response; HF, heart failure; HMOX, heme oxygenase; HRCR, Heart‐related circRNA; SIRT1, sirtuin 1; TGF‐β1, transforming growth factor‐β1; TPM4, tropomyosin‐4.

CircRNAs play an active role in myocardial fibrosis (Table 3). Upregulation of CircNFIB has been reported to inhibit adult fibroblast proliferation and reduce cardiac fibrosis by sponging miR‐433.^105^ Conversely, circHIPK3, which is expressed in the cytoplasm of fibroblasts, promotes fibroblast proliferation, migration and cardiac fibrosis by upregulating the expression of miR‐29b‐3p target genes.^106^ Meanwhile, circHIPK3 silencing exerted the opposite effect and improved cardiac diastolic function. Moreover, Wu et al.^107^ observed that circYap levels were significantly reduced in the hearts of patients with cardiac hypertrophy compared with patients with normal hearts. The injection of the circYap plasmid into mice attenuated cardiac hypertrophy and fibrosis while also improving cardiac function. Mechanistically, circYap promotes the binding of tropomyosin‐4 to gamma‐actin, resulting in the inactivation of actin polymerization and exerting an anti‐fibrotic effect. A recent study showed that circRNAs also play an essential role in drug‐induced cardiac fibrosis. Xu et al.^108^ demonstrated that circNlgn and its translation protein Nlgn173 mediate adriamycin‐induced cardiac fibrosis. The downstream factors of Nlgn173 can upregulate the expression of cytokines and the fibrosis regulators EGR1 and EGR3, which ultimately induce cardiac remodelling and fibrosis. With the help of RNA sequencing technology, Gu et al.^115^ and Chen et al.^116^ identified a considerable number of circRNA changes in cell and mouse models of cardiac fibrosis, respectively. Further research is needed to determine the specific functions of these circRNAs in cardiac fibrosis.

Additionally, excitingly, several studies have revealed the direct involvement of circRNAs in regulating heart failure (HF) and their potential as therapeutic targets (Table 3). For instance, CDR1as, a research hotspot, has been confirmed to be upregulated in the plasma of chronic HF patients and positively correlates with NYHA class in HF patients.^109^ Functional experiments in human cardiomyocytes confirmed that CDR1as could regulate the occurrence and progression of HF through the miR‐135a/heme oxygenase 1 and miR‐135b/heme oxygenase 1 pathways. Sun et al.^117^ analysed the microarray expression profile of circRNAs in the plasma of patients with chronic stable HF. Additional receiver operating characteristic (ROC) curve analysis showed that the changes in hsa_circ_0062960 had the highest sensitivity and specificity in HF patients and strongly correlated with serum brain natriuretic peptide (BNP) levels, suggesting its potential as a diagnostic biomarker for HF. Besides, epinephrine can upregulate the expression of circ‐HIPK3 through cAMP‐responsive element modulator (CREM), which in the long run promotes the progression of HF by increasing the concentration of Ca^2+^ in the cytoplasm of cardiomyocytes through the MiR‐17‐3p/adenylate cyclase type 6 (ADCY6) pathway.^110^ Zhang and Chen^111^ demonstrated that silencing circ_0062389 could alleviate cardiomyocyte apoptosis in HF rats through the TGF‐β1/Smad3 axis. Moreover, Zheng et al.^112^ explored the regulatory mechanism of circRNAs and iron metabolism in HF, demonstrated that circSnx12 regulates ferroptosis during HF by sponging miR‐224‐5p and constructing a circRNA‐miRNA–mRNA network, thus enriching the research of circRNAs in the field of HF.

## 14 ARRHYTHMIA

Atrial fibrillation (AF), the most common form of arrhythmia, is characterized by rapid and irregular electrical activation of the atrium, which can cause heart failure and is an important factor in ischemic stroke.^118^, ^119^, ^120^ To date, six studies have demonstrated significant changes in circRNA expression in the cardiac tissue of AF patients.^121^, ^122^, ^123^, ^124^, ^125^, ^126^ Zhang et al.^121^ established by RNA sequencing that 238 circRNAs were upregulated and 58 were downregulated in the left atrial appendage of patients with nonvalvular persistent AF compared with controls. Subsequent GO classification and KEGG annotation analysis showed that circRNA‐related ceRNA networks may affect AF through calcium and myocardial contraction alterations. Four additional studies found that 636, 280, 147 and 7566 circRNAs were dysregulated in AF patients’ cardiac tissue.^122^, ^123^, ^124^, ^125^ Since some patients in the study populations had comorbidities and the aetiology of atrial fibrillation is unclear, the number of circRNA changes varies among the studies. Hu et al.^126^ explored the expression profile of circRNAs in the atrial tissue of rheumatic heart disease patients with persistent AF and non‐AF patients with normal hearts. Compared with non‐AF patients with normal hearts, 108 circRNAs were differentially expressed in AF patients, of which 51 were upregulated and 57 were downregulated. Notably, the patients enrolled in this study had no other comorbidities.

Furthermore, two studies focused on whether circRNAs exhibited differential expression levels in various stages of AF.^127^, ^128^ In the first study, 83 (48 upregulated and 35 downregulated) and 99 (58 upregulated and 41 downregulated) circRNAs with significantly different expression levels were identified in paroxysmal AF and persistent AF compared with the control group, respectively.^127^ In the second study, the increased number of circRNA species detected was accompanied by miRNA downregulation during the transition from paroxysmal to permanent AF.^128^ Currently, research on the role of circRNAs in arrhythmia mainly focuses on AF. Nevertheless, most of these studies have been limited to the construction of circRNA‐miRNA–mRNA networks based on the identification of circRNA expression levels, and the specific molecular mechanisms of action remain to be elucidated.

## 15 HYPERTENSION

Several completed studies have confirmed the differential expression of circRNAs in hypertensive patients. For example, Wu et al.^129^ determined that the expression level of hsa‐circ‐0005870 in the peripheral blood of hypertensive patients was downregulated by circRNA array analysis and RT–PCR. By constructing a miRNA–mRNA network targeted by hsa‐circ‐0005870, the authors predicted that five miRNAs, including hsa‐miR‐6807‐3p, could become MREs of hsa‐circ‐0005870, which would help to reveal the potential mechanism of hsa‐circ‐0005870 involved in the regulation of hypertension. In a case‐control study, Bao et al.^130^ found that hsa_circ_0037911 was significantly elevated in blood samples from patients with essential hypertension (EH) compared with the non‐EH group. They also observed higher levels of hsa_circ_0037911 in the EH group in patients who were male, smoked and drank alcohol, which are risk factors for EH. In another case‐control study, He et al.^131^ identified that significantly elevated hsa_circ_0105015 in plasma was a risk factor of EH. Meanwhile, high expression of hsa_circ_0105015 combined with low expression of hsa‐miR‐637 may indicate vascular inflammation. Together, these studies have shown that circRNAs have the potential to become valuable molecular biomarkers for EH.

## 16 PULMONARY ARTERIAL HYPERTENSION

Pulmonary arterial hypertension (PAH) is a life‐threatening disease with progressively increased pulmonary arterial resistance due to pulmonary arterial proliferation and remodelling, ultimately leading to right heart failure and death.^132^, ^133^ The pathological mechanism of PAH is very complex, and multiple factors, such as inflammation, endothelial injury, proliferation, apoptosis and vascular remodelling, are involved.^134^ Two independent research teams first identified 351 and 64 differentially expressed circRNAs in blood samples from chronic thromboembolic pulmonary hypertension (CTEPH) patients and lung tissue from hypoxia‐induced PAH mice, respectively.^135^, ^136^ In a subsequent prospective cohort study, Zhang et al.^137^ observed significantly elevated serum hsa_circ_0068481 levels in patients with idiopathic pulmonary arterial hypertension (IPAH). Moreover, hsa_circ_0068481 strongly correlated with baseline clinical characteristics and clinical outcomes of IPAH patients. The canonical circRNA CDR1as can act as a sponge for miR‐7‐5p and play a crucial role in hypoxia‐induced HPASMC mineralization by upregulating calcium/calmodulin‐dependent kinase II‐delta (CAMK2D) and calponin 3 (CNN3).^138^ Based on the screening of differentially expressed circRNAs in CTEPH patients, another study used drug‐gene interactions to predict two candidate drugs suitable for treating CTEPH from the immunology perspective.^139^

Given the critical role of pulmonary artery smooth cell (PASMC) hyperproliferation in the pathological progression of pulmonary vascular remodelling in PAH, several studies have focused on the role of circRNAs in PASMC proliferation and differentiation. For instance, hsa_circNFXL1_009 was significantly downregulated in chronic obstructive pulmonary disease (COPD)‐induced PAH patients and hypoxia‐induced PASMCs, while overexpression of hsa_circNFXL1_009 attenuated hypoxia‐induced hPASMC proliferation and migration.^140^ Similarly, circ‐Sirt1 and circSMOC1 could effectively inhibit the abnormal proliferation and migration of PASMCs and improve pulmonary vascular remodelling in PAH rats.^141^, ^142^ However, circRNAs can also exert opposite effects. For example, circRNA‐calmodulin 4 (circ‐calm4) circ‐calm4 and circRNA‐glutamate metabotropic receptor 1 (circ‐Grm1) promote PASMCs proliferation and migration by regulating the circ‐calm4/miR‐337‐3p/myosin 10 signalling pathway and the circ‐Grm1/Grm1/repressor/activator protein‐1 (RAP1) axis, respectively.^143^, ^144^ Additionally, a recent study showed that CircSIRT1 was elevated in a rat model of PAH, while injection of the sh‐circSIRT1 lentiviral vector ameliorated PAH in rats.^145^ Mechanistically, the knockdown of CircSIRT1 inhibited proliferation, migration and autophagy in hypoxic PASMCs by regulating the miR‐145‐5p/Akt3 pathway. Overall, previous studies have confirmed that circRNAs play an important role in PAH (Table 4). However, the investigation of other mechanisms through which circRNAs affect PAH remains an area for future research.

**TABLE 4:** circRNAs associated with pulmonary arterial hypertension.

circRNA	Exp	Targets	Mechanism	Function	Ref
circ_0068481	↑	Unknown	Unknown	Circ_0068481 is valuable in the diagnosis and prediction of poor clinical outcomes in IPAH patients	[137]
CDR1as	↑	miR‐7‐5p	miRNA sponge	Induces PASMCs mineralization in hypoxia through miR‐7/CNN3/CAMK2D signal pathway	[138]
hsa_circNFXL1_009	↓	miR‐29b	miRNA sponge	hsa_circNFXL1_009 is protective in HPVR through the hsa‐miR‐29‐2‐5p‐KCNB1 axis	[140]
Circ‐Sirt1	↓	SIRT1 mRNA	Transcriptional regulation of parental Gene	Overexpression attenuates the progression of PAH	[141]
CircSMOC1	↓	miR‐329‐3p PTBP1	miRNA sponge Protein sponge	Overexpression alleviates pulmonary vascular remodelling	[142]
circ‐calm4	↑	miR‐337‐3p	miRNA sponge	Promotes PASMCs proliferation via circ‐calm4/miR‐337‐3p/myosin 10 axis	[143]
circ‐Grm1	↑	FUS	Protein sponge	Promotes the proliferation and migration of PASMCs via the circ‐Grm1/Grm1/Rap1 signal pathway	[144]
circSIRT1	↑	miR‐26b‐5p	miRNA sponge	Knockdown inhibits PASMCs autophagy through the miR‐145‐5p/Akt3 axis	[145]

Abbreviations: CAMK2D, calcium/calmodulin‐dependent kinase II‐delta; CNN3, calponin 3; GRM, glutamate metabotropic receptor; IPAH, pulmonary arterial hypertension; KCNB1, voltage‐gated potassium (K+) channel subfamily B member 1; PAH, pulmonary arterial hypertension; PASMCs, pulmonary artery smooth muscle cells; RAP1, repressor/activator protein‐1.

## 17 DIABETIC CARDIOMYOPATHY

Diabetic cardiomyopathy (DCM), the most severe complication of diabetes mellitus (DM), is characterized by a pathophysiological condition of abnormal myocardial structure and function in DM patients in the absence of cardiovascular risk factors such as coronary heart disease, hypertension and valvular heart disease.^146^ Recently, a study identified 29 upregulated and 29 downregulated circRNAs in the myocardial tissue of db/db mice with DCM. Subsequent functional analysis indicated that these differentially expressed circRNAs and their target mRNAs were mainly involved in the regulatory pathways of glycolipid metabolism. However, the specific pathway has yet to be further verified.^147^ Furthermore, Yang et al.^148^ found that hsa_circ_0076631, a novel circRNA named caspase‐1‐associated circRNA (CACR), was significantly elevated in both DCM cardiomyocytes and the serum of diabetic patients. FISH and luciferase activity experiments demonstrated that CACR acted as a ceRNA of miR‐214‐3p and downregulated its expression. CACR knockdown attenuated pyroptosis and downregulated the expression of the pyroptosis‐related factor caspase‐1 in DCM cardiomyocytes, while the knockdown of miR‐214‐3p reversed these effects. This result reflects that CACR can promote cardiomyocyte pyroptosis in DCM via the CACR/miR‐214‐3p/caspase‐1 axis.

Surprisingly, despite being extensively studied, CDR1as, a circRNA, was found to be upregulated in the myocardium of DCM mice.^149^ Meanwhile, CDR1as knockdown can inhibit cardiomyocyte apoptosis and improve cardiac function in DCM mice. Subsequent research demonstrated that the CDR1 transcription factor FOX3 upregulated CDR1 and activated the Hippo signalling pathway, which promoted cardiomyocyte apoptosis. These findings suggest that circRNAs could be a promising therapeutic target for DCM.

## 18 FUTURE PERSPECTIVES AND CONCLUSIONS

Cardiovascular diseases remain the leading cause of death from non‐infectious diseases worldwide. The current treatment for CVDs mainly focuses on controlling risk factors, early detection and diagnosis, individualized drug treatment and improved outcomes. Sensitive, efficient and convenient biomarkers greatly help in the prevention, diagnosis and treatment of CVD. Traditional biomarkers include cardiac troponin T (cTnT), creatine kinase isoenzyme, myoglobin, B‐type natriuretic peptide (BNP) and C‐reactive protein (CRP), which play a significant role in reflecting myocardial injury, cardiac function and the cardiovascular inflammatory state. However, the diagnosis and treatment of CVD remain unsatisfactory, and therefore, exploring novel biomarkers and potential therapeutic strategies is necessary.

Although research on circRNAs in CVDs is just emerging when compared with other research fields, such as cancer and neurological disorders, multiple studies have confirmed that circRNAs with stable, highly conserved and longer half‐lives have bright prospects as candidate biomarkers and treatment targets for CVDs. First, circRNA has a long half‐life and can be stably expressed in blood, making it easy to detect and an ideal diagnostic biomarker. Second, previous studies have confirmed that some circRNAs are dysregulated in various CVDs and even in different stages of the same disease, which helps to reflect the occurrence and dynamic progression of CVD. Third, artificial circRNA sponges (circmiRs) have been shown to attenuate the progression of stress overload‐induced cardiac hypertrophy and HF in mice by exploiting the function of miRNA sponges of circRNAs.^114^ Therefore, using exogenous circRNAs as therapeutic miRNA antagonists for the treatment of CVD is also a research field worthy of active exploration and has excellent prospects. Finally, the added value of circRNAs for the prognosis of CVD patients has been confirmed.^78^, ^79^ Future large‐scale, multicentre studies are helpful to further prove the feasibility of translation from bench to clinic. However, challenges remain in applying circRNAs for diagnosing and treating CVDs Compared with traditional biomarkers, the detection cycle of circRNAs is longer due to the relatively imperfect detection technology. How to improve the detection efficiency of circRNAs through technological innovation is also a question worth considering. Additionally, the current research on circRNAs in hypertension, DCM and arrhythmia other than AF is still in its infancy. Therefore, detailed and meticulous studies to clarify the role of circRNAs in these diseases may lead to breakthrough progress. In addition, numerous studies have used bioinformatics methods to construct a circRNA‐miRNA–mRNA interaction network, the specific mechanism still requires further research and verification.

Overall, although exploring circRNAs is only the tip of the iceberg, existing studies have shown that circRNAs have tremendous potential in preventing, diagnosing and treating CVDs. With the continuous progress of scientific technology and detection methods, we have reason to believe that gaining insight into the exact regulatory mechanism, complete regulatory network and specific clinical significance of circRNAs will boost our knowledge of circRNAs in the occurrence and development of CVDs and develop novel, circRNA‐dependent methods for the diagnosis, treatment and prognosis of cardiovascular diseases.

## AUTHOR CONTRIBUTIONS

**Chen Ding:** Writing – original draft (lead). **Yafeng Zhou:** Funding acquisition (lead); writing – review and editing (lead).

## FUNDING INFORMATION

This work was supported by grants from the National Natural Science Foundation of China (81873486), the Science and Technology Development Program of Jiangsu Province‐Clinical Frontier Technology (BE2022754), Clinical Medicine Expert Team (Class A) of Jinji Lake Health Talents Program of Suzhou Industrial Park (SZYQTD202102), Suzhou Key Discipline for Medicine (SZXK202129), Demonstration of Scientific and Technological Innovation Project (SKY2021002), Suzhou Dedicated Project on Diagnosis and Treatment Technology of Major Diseases (LCZX202132) and Research on Collaborative Innovation of medical engineering combination (SZM2021014). The funders had no roles in the study design, data collection, analysis, decision to publish or preparation of the manuscript.

## CONFLICT OF INTEREST STATEMENT

The authors confirm that there are no conflicts of interest.

## Data Availability

Data sharing is not applicable to this article as no new data were created or analyzed in this study.
